# Assessing the evolution of SARS-CoV-2 lineages and the dynamic associations between nucleotide variations

**DOI:** 10.1099/acmi.0.000513.v3

**Published:** 2023-07-20

**Authors:** Asmita Gupta, Reelina Basu, Murali Dharan Bashyam

**Affiliations:** ^1^​ Laboratory of Molecular Oncology, Centre of DNA Fingerprinting and Diagnostics, Hyderabad, India

**Keywords:** SARS-CoV-2 genome evolution, pathogenesis, vaccination breakthrough, COVID-19, mutational co-occurrence

## Abstract

Despite seminal advances towards understanding the infection mechanism of SARS-CoV-2 (severe acute respiratory syndrome coronavirus 2), it continues to cause significant morbidity and mortality worldwide. Though mass immunization programmes have been implemented in several countries, the viral transmission cycle has shown a continuous progression in the form of multiple waves. A constant change in the frequencies of dominant viral lineages, arising from the accumulation of nucleotide variations (NVs) through favourable selection, is understandably expected to be a major determinant of disease severity and possible vaccine escape. Indeed, worldwide efforts have been initiated to identify specific virus lineage(s) and/or NVs that may cause a severe clinical presentation or facilitate vaccination breakthrough. Since host genetics is expected to play a major role in shaping virus evolution, it is imperative to study the role of genome-wide SARS-CoV-2 NVs across various populations. In the current study, we analysed the whole genome sequence of 3543 SARS-CoV-2-infected samples obtained from the state of Telangana, India (including 210 from our previous study), collected over an extended period from April 2020 to October 2021. We present a unique perspective on the evolution of prevalent virus lineages and NVs during this period. We also highlight the presence of specific NVs likely to be associated favourably with samples classified as vaccination breakthroughs. Finally, we report genome-wide intra-host variations at novel genomic positions. The results presented here provide critical insights into virus evolution over an extended period and pave the way to rigorously investigate the role of specific NVs in vaccination breakthroughs.

## Data Summary

All the raw sequencing data generated and used in this study have been submitted to the Sequencing Read Archive (SRA) with project accession ID PRJNA691556. The custom R codes and associated data can be accessed from the Github repository (https://github.com/asmitagpta/nCov19-seq). The accession IDs of all the samples submitted to the GISAID database has been provided in Table S2. Supplementary material can be found in Figshare: https://doi.org/10.6084/m9.figshare.22293376.v3 https://doi.org/10.6084/m9.figshare.23697210.v1 [1].

## Introduction

In December 2019, a local outbreak of multiple cases of acute pneumonia [later classified as coronavirus disease 2019 (COVID-19, https://www.who.int/news-room/q-a-detail/coronavirus-disease-covid-19)] was reported in Wuhan, Hubei province, China, caused by a novel coronavirus, severe acute respiratory syndrome coronavirus 2 (SARS-CoV-2). The outbreak was soon followed by a rapid worldwide transmission which led to the World Health Organization (WHO) declaring COVID-19 as a global pandemic in March 2020. The rapid transmission rate of SARS-CoV-2 has resulted in 281 808 270 infections and >5 million deaths worldwide (as per WHO statistics, collected up to 29 December 2021). More importantly, 2021 witnessed the emergence of multiple virus variants [2, 3], of which five were classified as Variants of Concern (VoCs) by the WHO (https://www.who.int/en/activities/tracking-SARS-CoV-2-variants/) based on their higher transmissibility [4–7] and/or enhanced ability to escape neutralization by antibodies [8–13]. Of the five, the B.1.617.2 and B.1.1.529 lineages (designated, and hereinafter mentioned, as ‘Delta’ and ‘Omicron’, respectively, by the WHO) have exhibited maximum transmission during 2021, making them the predominant viral forms worldwide [14, 15]. Furthermore, a constant change in the frequencies of dominant viral lineages circulating in the population, arising from the accumulation of nucleotide variations (NVs) through favourable selection, is understandably expected to be a major determinant of disease severity and possible vaccine escape [16, 17]. Since host genetics is also expected to play a major role in shaping virus evolution [18], it becomes critical to study within-host diversity arising in the form of alternate alleles and corresponding genes harbouring them. Indeed, worldwide efforts have been initiated to identify specific virus lineage(s) and/or NVs that may cause a severe clinical presentation or facilitate vaccination breakthrough. As SARS-CoV-2 continues to evolve further into distinct lineages, with potentially increased pathogenicity and/or transmission abilities, it becomes imperative to study the emerging genomic variants, especially in the context of continual reports of possible immune escape and antibody neutralization imparted by a few NVs [17, 19].

The current study was initiated to analyse the sequences generated from SARS-CoV-2-infected samples (including 210 from our previous study), collected over an extended course of 19 months (from the beginning of the pandemic to October 2021) from the state of Telangana, India. We present the major trends of dominant lineages propagating in Telangana and corresponding trends in pan-Indian regions and worldwide, across this period. We also provide a comprehensive map of all NVs in the viral genome during this period and their possible association with vaccine escape. Finally, we have analysed specific virus genomic positions displaying intra-host diversity.

## Methods

### Sample collection strategy, dataset structure and features

A total of 3 543 samples [1 407 females and 2 091 males (information unavailable for 45 samples)], representing the period 1 April 2020 to 31 October 2021, from Telangana, India, were analysed in this study (Table S1A available in the online version of this article). The sample collection strategy for the period April 2020 to February 2021 was unchanged from our previous study [20]. For the subsequent period, nasopharyngeal/oropharyngeal swabs were collected from several reverse transciptase (RT)-PCR-based testing centres as well as multi-speciality hospitals across Telangana, as per guidelines established by the Indian SARS-CoV-2 Genome Consortium (INSACOG) [21]. In addition, samples received in the Covid-19 testing laboratory in CDFD, Hyderabad, were also included in the study. The work was initiated following approvals from the Institutional Bioethics committee and Biosafety committee. Sample collection peaked during the months of June and July 2020, followed by a hiatus, and subsequently increased from March 2021 onwards, roughly coinciding with the first and second waves of the pandemic, respectively. Of the total cases, 360 represented the age group <18 years, 2 674 represented the age group 18–60 years and 55 represented the age group >60 years, while age was not documented for 154 cases. The dataset also comprised two independent sets of cases belonging to local isolated transmission clusters (so-called ‘super-spreader’ events) (Table S1A).

Cases were classified as vaccination breakthroughs if they reported infection ≥14 days after receiving a second dose of either ChAdOx1 [22] (commercial name Covishield, based on recombinant, replication deficient chimpanzee adenovirus vector, developed at Oxford University, Oxford, UK) or BBV152 [23] (commercial name Covaxin, whole virion inactivated Vero cells developed by Bharat Biotech Ltd, India) vaccine or ≥21 days of receiving a first dose [22] [24] [25]. The dataset included a total of 313 vaccination breakthrough cases, of which 244 came from Telangana, 18 Uttar Pradesh (obtained from the Banaras Hindu University) and 51 to Chennai, Tamil Nadu (obtained from the Department of Public Health and Preventive Medicine, State Public Health Laboratory) (Table S1B, S1C). Of these, 154 were completely vaccinated and 149 had received only one vaccination dose, while the status of 10 was unavailable. The majority of cases (228/313; 72.8 %) received the ChAdOx1 vaccine, while a small proportion (36/313; 11.5 %) received the BBV152 [23] (Table S1 A–C). Vaccine identity was unknown for 49 samples. The vaccination dates were unavailable for 12 partially vaccinated and two completely vaccinated cases and therefore these 14 samples were excluded during NV analysis of vaccination breakthrough cases

For pan-India and worldwide analyses of widespread lineages, a dataset comprising 31 546 (India) and 678 438 (world) consensus genomes (*fasta* files) for the period March 2021 to October 2021 were accessed from the publicly available Global Initiative on Sharing All Influenza Data [26] (GISAID, https://www.gisaid.org/) repository; accession IDs for all the sequences submitted to GISAID from this study are listed in Table S2.

### SARS-CoV-2 RNA extraction and sequencing

Total RNA was isolated in a Biosafety level 2+ (BSL-2+) environment following standard protocols using the RNA isolation kit (MagRNA-II viral RNA extraction kit, Cat. No. G2M030620; Genes2Me; molecular RNA extraction kit, Cat. No. COVEX 100PS; Q-lineBiotech; nucleic acid extraction kit, Cat. No. A200-96; Zybio Inc.) as per the manufacturers’ instructions. The RNA extraction and sequencing protocol has been described in our previous study [20]. Briefly, each RNA sample was subjected to RT-PCR for Envelope (E) and RNA-dependent RNA polymerase (RdRp) genes using the nCoV-19 RT-PCR detection kit (Cat. No. NCoV-19ER100PS; Q-lineBiotech) or the ViralDetect-II multiplex real-time PCR kit for COVID-19 (which also detected the N-gene; Cat. No. G2M020220; Genes2Me). As mentioned in our previous study, since RdRp consistently provided more robust amplification than the E-gene, we considered Ct (threshold cycle) values of RdRp alone for analysis. Samples exhibiting a Ct value of <30 (E and RdRp genes) were selected for whole genome sequencing.

The isolated RNA was reverse transcribed using random primer mix (New England Biolabs) and Superscript-IV (Thermofisher Scientifi). The synthesized cDNA was amplified using a multiplex PCR protocol, producing 98 amplicons across the SARS-CoV-2 genome (https://artic.network/, primer version V3, https://www.protocols.io/view/ncov-2019-sequencing-protocol-v3-locost-bp2l6n26rgqe/v3). The amplified products were processed for tagmentation and indexing PCR for Illumina Nextera UD Indexes Set A, B, C, D (Illumina) (384 indexes, 384 samples). All samples were processed as 96-well plate batches that consisted of one each of COVIDSeq positive control HT (CPC and, quantified (Qubit 2.0; Invitrogen) and fragment sizes were analysed in Agilent Tapestation 4200 (Agilent Technologiess). The pooled library was further normalized to 10 nM concentration and 10 µl of each normalized pool containing index adapter set A, B, C and D was combined in a new microcentrifuge tube to a final concentration of 2 nM. The pooled libraries were denatured and sequencing was performed on a Nextseq 2000 using the P2 100 Cycle kit with 1×101 bp sequencing chemistry. About 50–100 Mb of data were generated for each sample.

### *In silico* workflow for processing genome sequencing data

The raw sequencing data in *fastq* format were subjected to quality checks including filtering low-quality reads, determination of sequencing depth and adapter trimming using Trimmomatic [27]. All reads shorter than 30 bases or with an average Phred quality score <20 were discarded. Eighteen samples were rejected because of low overall sequencing depth and poor quality. The trimmed reads were then aligned to the reference Wuhan sequence (NCBI ID NC_045512.2) using the bwa-mem [28] algorithm. Post-alignment filtering and quality assessment was performed using samtools [29]. Single nucleotide variants (SNVs) were identified using iVar [30] which works on the *mpileup* output from samtools. The variants were annotated using SnpEff [31] and further filtered to remove all problematic sites documented to be prone to accumulate sequencing errors by multiple sources as recommended earlier (https://virological.org/t/issues-with-sars-cov-2-sequencing-data/473) [32]. Sequences with high coverage, low N content and associated with complete metadata were included for further analysis. Reads were assembled to generate consensus *fasta* file using samtools mpileup and the consensus module of iVar with a base assigned as consensus if it had a minimum depth of at least 10 reads (setting ivarMinDepth=10). Sequences where the N content was >30 % and sequence length <27 000 were rejected. Lineage assignment was done on consensus *fasta* files using Pangolin [33] v3.1.16.

All alleles with an allele frequency of >90 % were classified as NVs. For analysis of NV cross-correlation, a methodology similar to the one reported in our previous study [20] was used. Briefly, a binary matrix was constructed for each sample with all NVs found in >5 % samples as columns, indicating whether an NV of interest was present or absent in a sample. NVs exhibiting low standard deviation across samples were not included in the analysis. Pairwise Pearson correlation coefficients were estimated for this binary matrix using the *cor* function in R. *P*-values indicating the significance of association between each pairwise correlation coefficient was estimated using the *cor.test* function with *chi-square* test. This matrix was then used to visualize the NV cross-correlation maps in R with the *corrplot* function [34]. Odds ratios for estimating the association likelihoods of genomic alterations with vaccination breakthrough cases were estimated by creating contingency matrices for each NV identified in >5 % of vaccinated samples and were compared with multiple random subsamples of non-vaccinated cases from March 2021 onwards.

The estimation of intra-host single nucleotide variations (iSNVs) was carried out using Lofreq [35], a variant caller with a high sensitivity to predict iSNVs with an allele frequency as low as 1 % [35]. Minor alleles with allele frequencies between 2 and 50 %, and minimum sequencing depth of 100×, were classified as iSNVs, and were used for further analysis.

All statistical tests and analyses were performed using custom R scripts. All structural representations were generated in PyMOL (The PyMOL Molecular Graphics System, Schrödinger, LLC).

## Results

### B.1.617.2 (‘Delta’) displaced all previously circulating lineages from March 2021 onwards

**Fig. 1.**
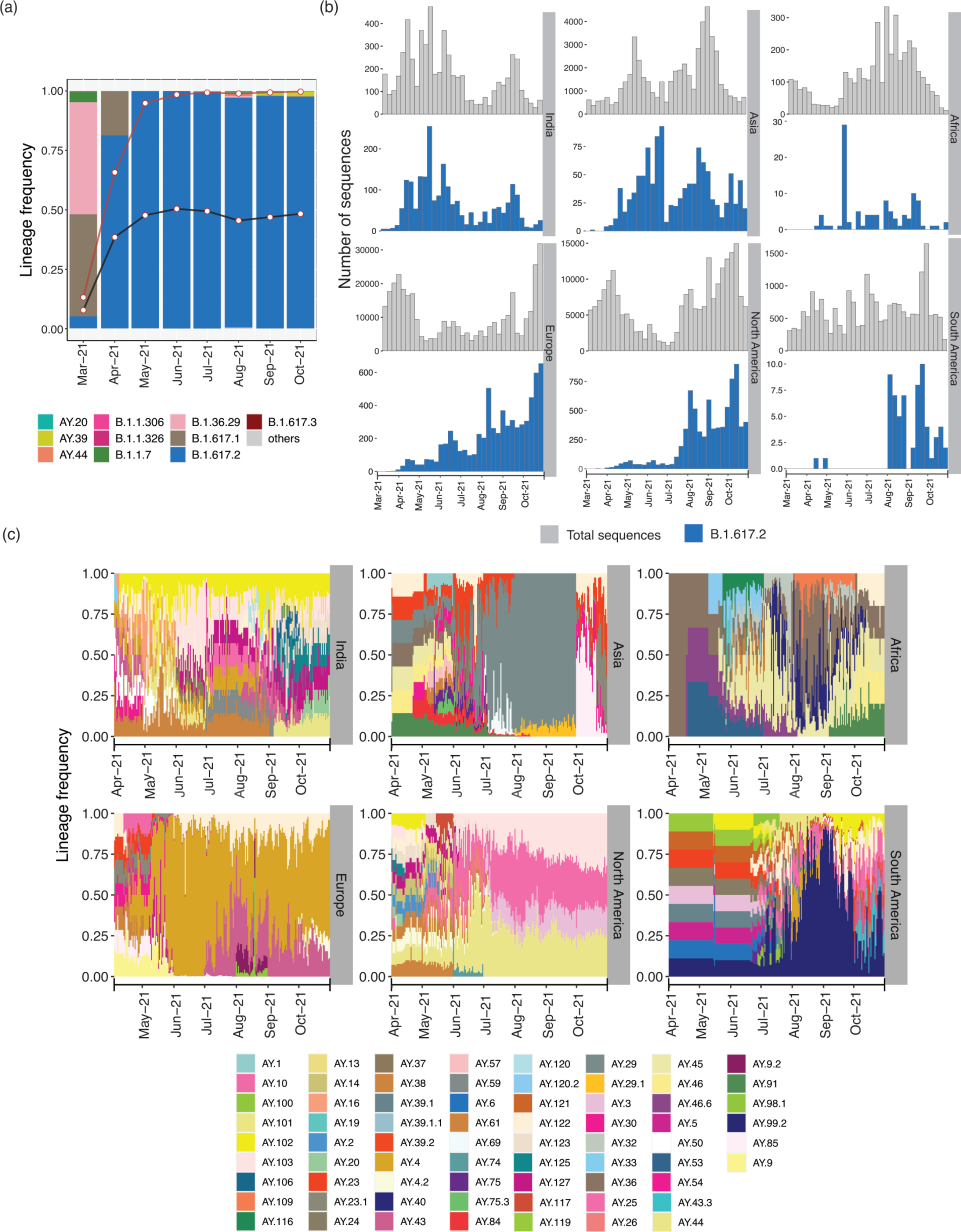
Comparative timeline of major SARS-CoV-2 lineage distribution in Telangana, India. (**a**) Frequency distribution of the major lineages per month from March 2021 (a complete timeline starting from April 2020 is provided in Fig. S1a). The black and red lines show the frequency distribution trend of Delta and Delta plus its sub-lineages in India (excluding Telangana), respectively; white points show the corresponding monthly frequency. (**b**) Timeline of Delta variant distribution (blue) and total (grey) cases in India and rest of the world (all data were obtained from GISAID). (**c**) Timeline of changing frequency of Delta sub-lineages across the world. Only those sub-lineages present in >5 % of total sequences submitted from the region were included. In (b) and (c), while estimating frequencies for the Asian countries, Indian sequences were excluded.

We identified a clear shift in the dominant lineages present in Telangana, India, from 2020 to 2021 (Figs 1a and S1a; the complete distribution of all lineages in the dataset is provided in Table S3). The B.1.1.306 and B.1.1.326 lineages (both detected first in India and considered to have transmission links to Zambia, Somalia and Bahrain (https://cov-lineages.org/lineage_list.html) were dominant from May 2020 to September 2020. The period October 2020 to March 2021 witnessed an upsurge of B.1.36.29 (assigned as ‘Indian Lineage’ by Pangolin [33] and pangoLEARN [36]). December 2020 witnessed the emergence of the Kappa (B.1.617.1) lineage in the state population, and was present until April 2021. However, ‘Alpha’ (B.1.1.7), which was the first lineage classified by WHO as a VoC, appeared in the state population in February 2021 and was identified in samples until March 2021, consistent with other reports [37]. Spread of the Alpha lineage was higher and lasted longer in northern India, appearing as early as January 2021, and present until May 2021, while southern India witnessed a higher prevalence of the Kappa variant (B.1.617.1) (Fig. S1a,b). However, from March 2021 onwards, the Delta variant (B.1.617.2) constituted the major lineage detected in the state, replacing all other virus lineages circulating previously, indicative of its massive spread (Figs 1a and S1a). As expected, the extent of its spread in Telangana almost paralleled its surge in the rest of the country [38] (Fig. S1b). Lineage analyses on the two sample sets representing local transmission clusters (Table S1a) did not reveal enrichment of a specific lineage compared to other samples analysed from the same period in parallel (data not shown). The spread of Delta in India pre-dated its spread in other countries (Fig. 1b). Following its gradual rise in Asia, the Delta lineage spread to Europe, North America and South America, with the highest spike in June–August 2021 (Fig. 1b). By the end of June 2021, Delta was the major lineage in all geographical regions of the world [21].

In July 2021, the Delta variant was further classified (by Pangolin) into several sub-lineages (https://cov-lineages.org/) indicative of appearance of additional NVs (https://outbreak.info). From July 2021 onwards, AY.20, AY.39 and AY.44 were the major Delta sub-lineages observed in Telangana (Fig. 1a), though the fraction of Delta sub-lineages was higher in most Indian states compared to Telangana (Fig. S1b). Moreover, the major sub-lineages in other states varied from July 2021 onwards (Fig. S1b).

The distribution of Delta sub-lineages also differed worldwide (Fig. 1c). From mid-April 2021 onwards, AY.4 was most prominent in Europe, followed by AY.43 from July 2021; by contrast, AY.103, AY.44, AY.3 and AY.25 were more common in North America from June 2021. The sub-lineage AY.29 was highly prevalent in Oceania from July 2021 (Fig. S1c), while Asian countries (excluding India) displayed a mix of various sub-lineages, with AY.29 becoming prevalent between June and September 2021 (Fig. 1c). Similar to the distribution pattern observed in India, a mix of different Delta sublineages including AY.36, AY.40, AY.45, AY.46 and AY.91 was observed in sequences from Africa during May–October 2021. Given the consistently increasing repertoire of Delta sub-lineages, constant lineage re-assignment by Pangolin and the fact that a mere assessment of the lineages per se may be insufficient to understand the complete catalogue of SARS-CoV-2 NVs, we performed a detailed analysis of NV profiles obtained from the samples.

### Genome-wide analysis reveals a sudden change in the landscape of SARS-CoV-2 NVs coinciding with the second wave

**Fig. 2.**
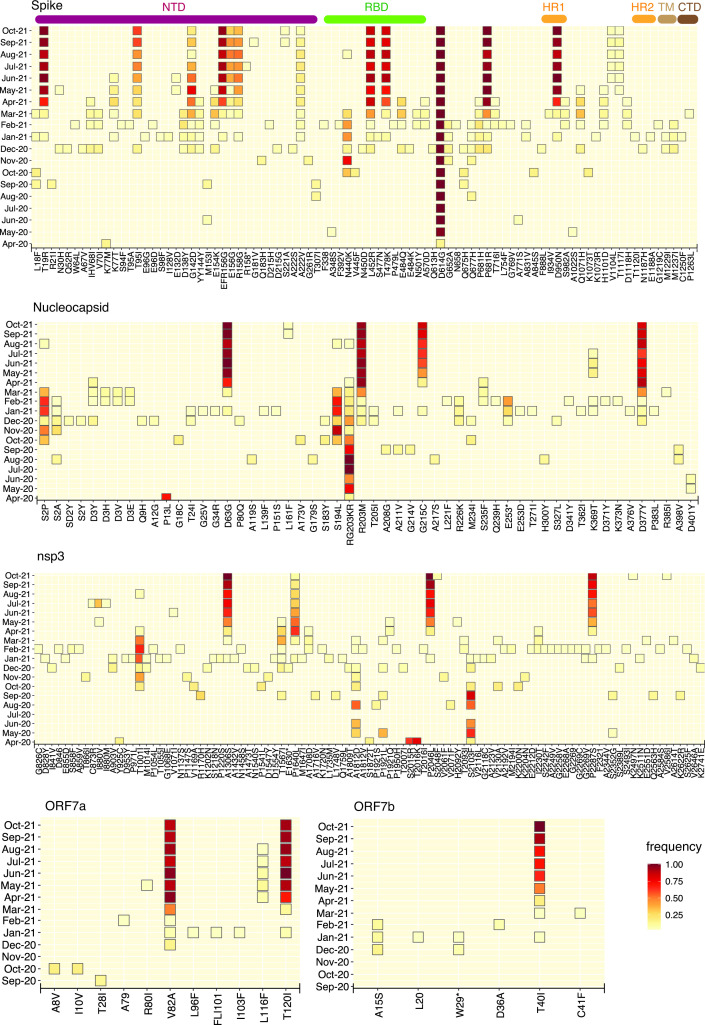
Monthly timeline of SARS-CoV-2 NV frequencies; variants present in <3 % of samples were excluded. Key domains in the S protein (first panel) are indicated at the top: NTD, N-terminal domain (brown); RBD, receptor binding domain (green); S2 subunit containing SD1 (subdomain 1), SD2 (subdomain 2) and S1/S2 cleavage sites (orange); CTD, C-terminal domain (black). NV frequencies for N protein (second panel), nsp3 (third panel), and ORF7a and ORF7b (bottom panels) are also shown.

Study of NVs not included in the NV signature of a lineage but that nevertheless could have arisen either due to positive selection or genetic drift may be critical from the perspective of public health surveillance. A monthly assessment revealed a significant shift in the NVs observed in Telangana during the course of the previous year (Fig. S2). Overall, a few NVs were consistently present in the population starting from April 2020, namely A23403G (D614G, S), C241T, C3037T (P924F, ORF1a: nsp3) and C14408T [P4720L (alternatively referred to as P323L), ORF1b: nsp12] (Fig. S2). Genes coding for envelope and membrane proteins (E and M genes, respectively) and the accessory protein ORF6 remained relatively free from high-frequency NVs until October 2021. The genomic landscape of the virus was marked by the presence of a few high-frequency NVs in the period from April 2020 to July 2020 (Fig. S2). The period from August 2020 to February 2021 witnessed the appearance and subsequent disappearance of many moderately frequent NVs, especially in ORF1ab. However, from March 2021 onwards, a larger number of NVs became evident at moderate to high frequencies throughout the genome (Fig. S2). Specifically, the S protein, nucleocapsid (N), ORF7a/b and nsp3 accumulated several missense NVs (Fig. 2). The S protein NVs included T19R, T95I, G142D, del156-157, A222V, L452R, T478K, D614G, P681R and D950N, along with the ubiquitous D614G (Fig. 2). With the exception of T95I, G142D and A222V, all other NVs were consistently present in >75 % of samples until October 2021 and were further associated with the appearance of the Delta lineage in the population (Fig. S3). Beginning in May 2021, additional NVs appeared in the S protein, which resulted in bifurcation of Delta into various sub-lineages (Figs 2 and S3). Notably, the frequency of T95I was highest in AY.20 compared to Delta and other sub-lineages while the frequency of G142D was highest in the Kappa lineage (B.1.617.1) compared to other lineages observed thereafter. A222V was another alteration shared by both Delta and AY.44 though it was present in higher frequencies in sub-lineage AY.44. Interestingly, an observation which clearly stood out from this analysis is that sub-lineages AY.20, AY.39 and AY.44 harboured several NVs [including M153I, A243P, L244F, V1104L and V1176F (in AY.20), S221A and A1080S (in AY.39), G181V (AY.20, AY.39), and T1117I and D1260E (in AY.44)] in the N-terminal domain (NTD), receptor binding domain (RBD) and the region between the two heptapeptide repeat sequences HR1 and HR2, which were either absent or present in lower frequencies in Delta (Fig. S3).

**Fig. 3.**
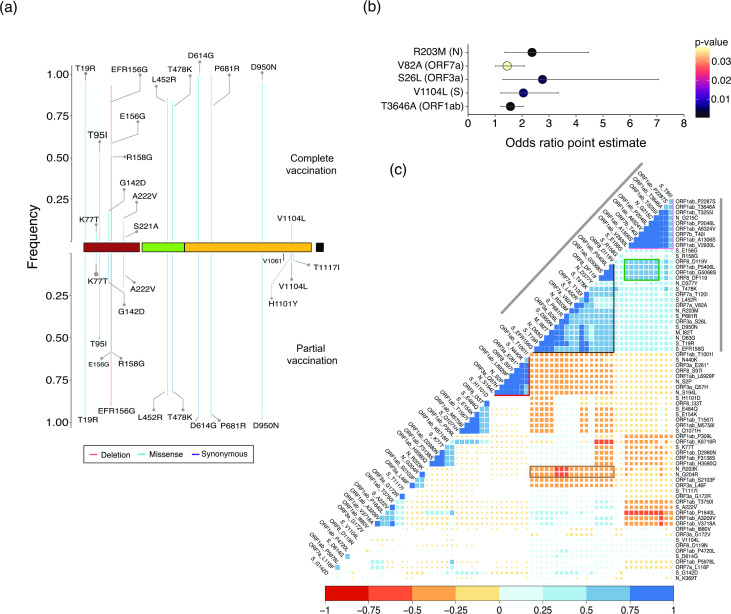
NV frequency in vaccinated cases and pairwise cross-correlation analysis. (**a**) Frequency of S protein amino acid alterations present in >3 % of completely (top, calculated for a total of 152 samples) and partially (bottom, total of 137 samples) vaccinated cases. (**b**) Odds ratio indicating the extent of association of specific variants with vaccination breakthrough cases. (**c**) Pairwise cross-correlation plot between all non-synonymous missense NVs present in >3 % of all the SARS-CoV-2 samples identified from Telangana, India, during April 2020 to October, 2021; the size of all coloured ‘squares’ is inversely proportional to the corresponding *P*-value of the correlation. Positions with *P*>0.05 appear as blank (or white). The colour key for positive (blue) and negative (red) correlation is given below the plot.

A striking observation in the N protein was the disappearance of RG203KR (triplet 28881-3GGG>AAC) from January 2021 onwards (Fig. 2). We observed the simultaneous appearance of R203M in Alpha as well as in Delta and its sub-lineages. Furthermore, the Delta lineage was marked additionally by N protein NVs D63G, G215C and D377Y. More importantly, several N protein NVs were restricted to Delta sub-lineages (being absent in Delta itself) including L161F, K361Q and K369T (AY.39), and A252S (AY.20) (Fig. S3).

Among the non-structural proteins that constitute ORF1a/b, nsp3 is the largest, and exhibited an increase in frequency of several NVs (I880V, A1306S, P1640L, P2046L, P2287S) from March, 2021, most of them being associated with Delta and its sub-lineages (Figs 2 and S3). The accessory proteins 7a and b, relatively free of NVs until December 2020, accumulated distinct high-frequency NVs including V82A (7a), T120I (7a) and T40I (7b) from January 2021 (Figs 2 and S3). Similarly, the NV landscape of ORF3a was significantly altered post-December 2020 and ORF1b:nsp12 (RdRp) accumulated the G5068S NV in addition to the ubiquitously present P4720L (Fig. S4). The frequencies of all these NVs in ORF7a/b, ORF3a and nsp12 were consistently high in samples classified as either Delta or its sub-lineages AY.20, AY.39 and AY.44, potentially reflecting an extended genomic variation footprint not observed in previous lineages (Figs S3 and S4). Furthermore (and similar to the observations made with respect to lineages), an inspection of the NVs in the samples collected from two local ‘super-spreader’ events (Table S1A) did not reveal enrichment of any specific NVs.

In a nutshell, the Delta sub-lineages were marked by the presence of several NVs, over and above those that defined the Delta lineage. Second, AY.20 and AY.39 had relatively higher numbers of NVs compared to other sub-lineages observed in the state. Since the emergence of vaccine breakthrough cases coincided roughly with the emergence of Delta and its sub-lineages, we further investigated whether the lineages or the NVs associated with them showed a higher likelihood of occurrence in vaccination breakthrough cases.

### Association of specific genomic NVs with vaccination breakthrough cases

A large fraction (70 %) of vaccination breakthrough cases belonged to the Delta variant, followed by its sub-lineages AY.44 (5.2 %), AY.20, AY.43 and AY.39 (5 % each) (Fig. S1d). Interestingly, 57, 39, 29 and 22 % of all samples classified as AY.32, AY.43, AY.35 and AY.20 respectively, belonged to vaccination breakthrough cases (Fig. S1d). We next analysed all S protein NVs present in >3 % of vaccinated cases. T19R, deletion EFR156G (156-157del), L452R, T478K, D614G, P681R and D950N, present in high frequency in the total dataset, were also present with highest frequencies (>80 %) in vaccinated cases (Fig. S5), as expected. In addition to these, T95I, which was present in slightly higher frequencies in samples belonging to AY.20 compared to Delta, was found in 50.8 % of all vaccinated samples. Another S protein alteration, V1104L (located in the S2 subunit), was present in ~7.5 % of all vaccinated cases. Intriguingly, the frequency of this NV was higher in AY.20-associated samples than in Delta itself (Fig. S3). Separate analyses of partial and completely vaccinated cases identified S221A (2.3 % cases) as an exclusive event in completely vaccinated cases (Fig. 3a). Similarly, T1117I (~3.9 % cases) and H1101Y (~1.9 % cases) were identified exclusively in partially vaccinated cases (Fig. 3a). We computed odds ratios to estimate the significance of association of genome-wide NVs with vaccination breakthrough cases. V1104L (S), S26L (ORF3a), V82A (ORF7a), R203M (N) and T3646A (ORF1ab) exhibited odds ratios of 1.5–3.0 [*P*<0.05, at 95 % confidence interval (Fig. 3b); details of upper and lower confidence interval limits are provided in Table S4], thereby providing further support towards favourable occurrence of these NVs in vaccination breakthrough cases

### NV cross-correlation maps reveal presence of extended NV signatures associated with different lineages

An NV (missense only) cross-correlation map arranged by hierarchical clustering revealed several clusters highlighting the frequency of co-occurring NVs within samples. The first major cluster immediately apparent from the cross-correlation map (Fig. 3c, grey lines at the sides) was formed by an extensive set of co-occurring NVs that highlight the Delta lineage (https://outbreak.info/situation-reports/delta). Within this large cluster, however, we identified the presence of two smaller (sub) clusters. One of these (black outline; Fig. 3c) encompassed S protein NVs P681R, L452R, T478K, D950N and T19R which were observed in high frequency in samples belonging to the Delta lineage (Fig. S3). This sub-cluster additionally displayed enriched co-occurrence of NVs in other genes, namely T120I, V82A (ORF7a), S26L (ORF3a), del 119/120 (ORF8), I82T (M) and D63G and D377Y (N), indicative of a potential extended genomic signature of the Delta lineage. Interestingly, this sub-cluster was mutually exclusive with another set of NVs [N440K (S), S2P, S194L (N), Q57H, stop gained E261* (ORF3a)] that were part of the B.1.36.29 lineage (Fig. 3c, the lineage defining NVs highlighted by a red outline) which was abundant before the emergence of Delta as mentioned above (Figs. 2 and S3). The Delta-defining cluster was also negatively correlated to the double amino acid changes in N protein R203K and G204R (brown rectangle; Fig. 3c) that was prevalent before the emergence of Delta, indicating a completely unique signature formed by NVs in Delta, mutually exclusive to that of previously circulating viral lineages in the population.

The second sub-cluster (pink outline; Fig. 3c) included T95I (S), G215C (N), T40I (ORF7b) and several NVs located in ORF1ab including A1306S, P2046L, P2287S (nsp3), T3255I (nsp4), T3646A [nsp6; also favourably associated with vaccination breakthrough cases (Fig. 3b)] and A6324V (nsp14). These NVs were found to be present in a large proportion (>90 %) of samples associated with Delta sub-lineages AY.20, AY.39 and AY.44 (Fig. S3). Though these NVs also exhibited positive correlation with the NVs in the Delta sub-cluster (Fig. 3c; black outline) a stronger correlation among the NVs within this new cluster points to a divergent evolution of these sub-lineages in the population. Furthermore, several NVs associated within this Delta sub-lineage sub-cluster displayed positive associations with a set of NVs formed by ORF8 (D119V, del 119/120) and ORF1ab (P5406L, G5068S) (green rectangle; Fig. 3c), reflecting an additional set of co-occurring common NVs occurring in Delta sub-lineages (AY.20, AY.29, AY.44) (Fig. S4).

**Fig. 4.**
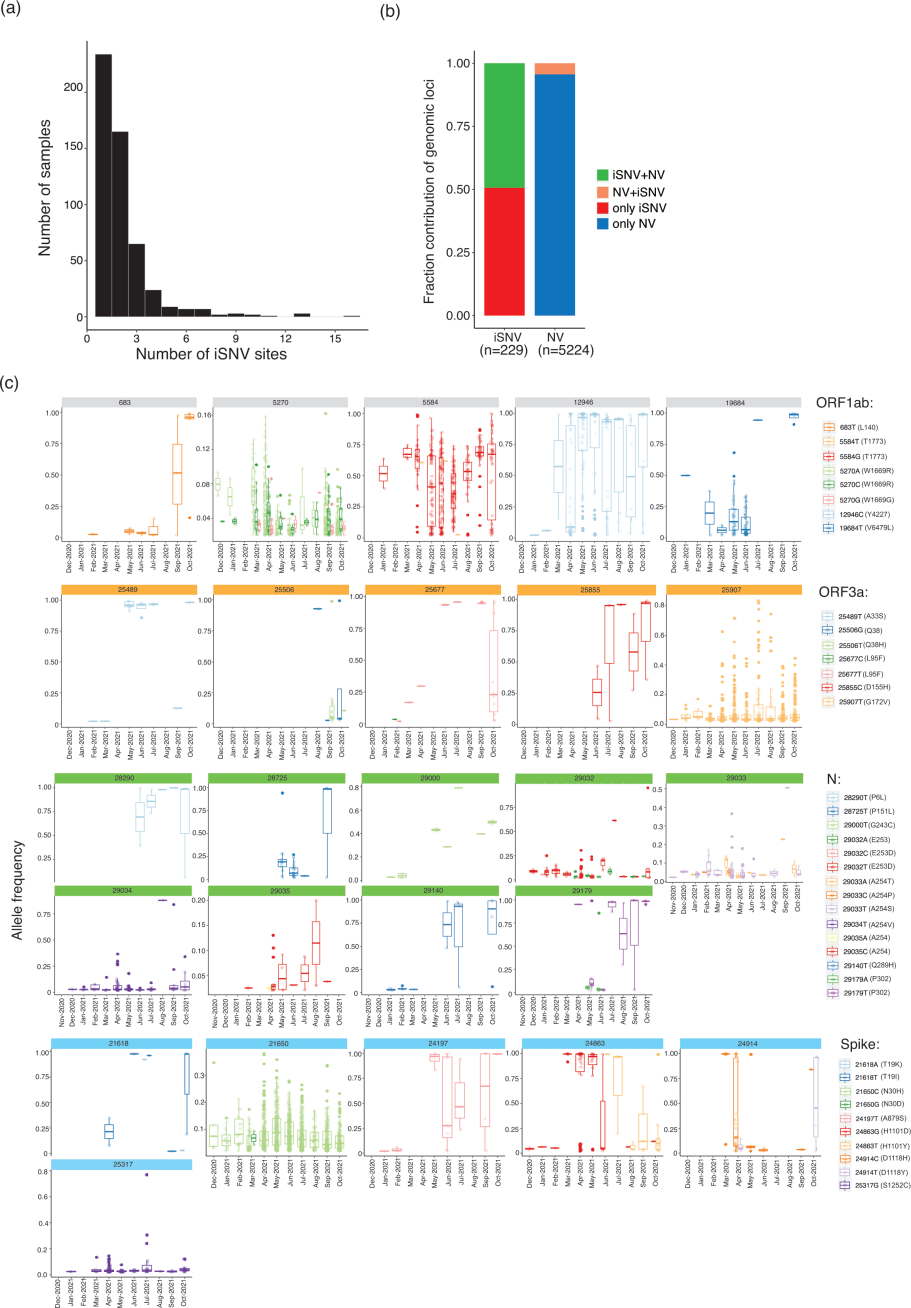
SARS-CoV-2 iSNVs identified from Telangana, India. (**a**) Distribution of number of iSNVs in samples. (**b**) Distribution of genomic loci with shared and unique iSNVs and NVs. (**c**) Timeline of allele frequency changes in the minor alleles in ORF1ab, ORF3a, N and S proteins. Box plots indicate allelic frequency distribution while points represent samples in which the allele was identified. Only those alternate alleles whose allele frequencies were either consistent or increased during the indicated timeline are shown.

We extended the analysis to vaccine breakthrough cases and observed a positive cross-correlation between S26L (ORF3a), P4720L (ORF1ab) and D614G (S) (correlation coefficient *r* ^2^>0.75) which was absent in non-vaccinated cases (Fig. S6). Interestingly, S26L (ORF3a) also exhibited highly significant association with vaccination breakthrough cases (Fig. 3b). Another positive cross-correlation was observed among V82A (ORF7a) and S protein NVs, L452R, T478K (*r* ^2^>0.75, as opp and, T95I (*r* ^2^ >0.5; <0.5 in non-vaccinated cases). Finally, another instance of increased positive correlation in vaccination breakthrough cases was formed by R203M (N), V82A and T120I (ORF7a) compared to non-vaccinated cases (Fig. S6). This aligns well with our previous observation, where both R203M and V82A displayed favourable odds of occurrence in vaccination breakthrough cases (Fig. 3b). Overall, the findings suggest that the genomic alterations in the S protein (L452R, T478K, P681R) which have previously been reported to increase escape from neutralizing antibodies and potentially associate with vaccination breakthroughs [8, 11] might arise in tandem with genomic changes located in non-S genes which are involved in modulating the downstream processes in viral life cycle (ORF1ab, N) and regulating the host immune response (ORF7a/b, ORF3a), thus increasing the probability of virus survival and causing breakthroughs.

### SARS-CoV-2 exhibits genomic plasticity as multiple sites contribute to generation of intra-host variants

Since virus genomic diversity arises within a host, it becomes important to uncover minor alleles originating in the form of iSNVs. A total of 545 samples (15.4 % of the total dataset) exhibited iSNVs, of which a majority [234; 43 % (6.6 % of the total dataset)] harboured minor allelic variants at a single genomic position and 165 samples [30% (4.6 % of the total dataset)] exhibited iSNVs at two positions (Fig. 4a). Interestingly, 11 samples contained more than nine sites with minor alleles. However, the Ct values in seven of these 11 cases were >22 (Table S5) indicating possible false calls due to sequencing error(s), as reported earlier [39–41]. The presence ofmore than nine iSNV sites in the remaining three samples could probably reflect mixed infections, as reported earlier [39]. N, nsp11, nsp9 and ORF3a exhibited a higher frequency of iSNVs (when normalized to gene size) compared to other proteins (Table S6).

We next endeavoured to map the co-occurrence of NVs and iSNVs in the genome. Across the 545 samples, a total of 229 genomic loci were involved in iSNVs, of which 113 loci were found to also share NVs (Fig. 4b). This observation stands in contrast to the total number of genomic loci which formed NVs across the dataset (5224), of which only 229 sites shared iSNVs. To investigate whether any iSNV site(s) which shared NVs coincided with mutation ‘hotspot’ region(s), we mapped all genomic positions which shared iSNV and NVs and estimated the sample frequencies of major and alternative alleles at these loci (Fig. S7). The analysis revealed that a very few [9053 (nsp4), 11201, 11332, 11 418 (nsp6), 21618, 23403, 23 604 (S), 26 767 (N) and 28 881 (N)] shared sites exhibited NVs (major alleles) which were widespread (sample frequency >45 %) in the dataset, suggesting that these loci could be mutation hotspots.

Another critical and arguably more important insight that iSNV analysis provides is by enabling effective temporal tracking of a novel minor allele from the time of its emergence. This can potentially identify an important NV before it becomes widespread in the population, and hence being of great benefit for public health surveillance. To this end, we evaluated changes in sample and allele frequencies of minor alleles identified in iSNV sites (Fig. 4c). The results revealed few iSNVs in ORF1ab (683T, 5584G, 12 946C and 19 684T), ORF3a (25 489T, 25 506T, 25 506G, 25 677T, 25 855C and 25 907T) and multiple positions in N (28 290T, 28 725T, 29 000T, 29 032T, 29 034T, 29 140T and 29 179T) where the sample and allele frequencies consistently increased from the time of their emergence (Fig. 4c). Allele frequencies of minor alleles at all these iSNV positions (with a few exceptions discussed below) reached 80 % over time, attesting to a transition from iSNV to NV. Furthermore, most of these iSNVs emerged in January 2021 (with a few emerging in December 2020 or February 2021), indicating a possible escape from a transmission bottleneck. A few notable examples include 25 907T (leading to 172V in ORF3a, present in 53 % of the total samples) and 29 034T in N (leading to 254V, present in 24 % of all samples).

In addition, a few iSNVs exhibited a significant increase in sample frequency across a long time period, despite exhibiting an allele frequency of <50 % (Fig. 4c). A notable example was all alternative alleles at position 5270 (nsp3), which exhibited an allele frequency <16 %, but were nonetheless detected in a significant fraction of samples (across the 3 543 samples, 5270A in 33 % cases, 5270C in 20 %, 5270G in 5 %) (Fig. 4c). Another such instance was observed at position 29 033 (N; 29033T in 6 %, 29 033A in 1.2 %) (Fig. 4c).

We next focused on S protein iSNVs, expected to be important in shaping virus transmission and immune escape. In addition, S protein iSNVs are reported to be rare events due to evolutionary constraints with many becoming lost, attesting to a narrow transmission bottleneck [42]. A few minor alleles, 19I (C21 618), 30H (A21 650), 879S (G24 197), 1101Y (C24 863), 1118Y (C24 914) and 1252C (C25 317), which were first observed in independent samples in January 2021, were consistently present in samples until October 2021 as well (sample distribution and allele frequency shown in Fig. 4c). Among these, the S protein iSNV-associated minor alleles that exhibited an increased sample frequency from the time of their emergence were 30H, 1101Y and 1 252C (the distribution of their allelic frequencies in shown in Fig. 4c). However, the allelic frequencies of 30H and 1 252C, in all the samples in which they were detected, was <50 % (with the exception of one sample which harboured the 1252C allele at an allele frequency >75 %). By contrast, from June to September 2021, the sample frequency of 1101Y increased from 0.6 to 3 %, with a concordant increase in corresponding allele frequency (Fig. 4c). Interestingly, the H1101Y NV was also present in >5 % of all partially vaccinated samples. Subsequent analysis on the potential functional impact of these iSNVs is currently underway.

## Discussion

SARS-CoV-2 continues to cause significant morbidity and mortality worldwide, making it important to perform regular genomic surveillance to detect emerging virus NVs. The estimated mutation rate of SARS-CoV-2 is about 1.1×10^−3^ substitutions per site per year [43]. The constant virus evolution leading to emergence of new variants is shaped by several factors including host genetics and immune response [44, 45], strong selection pressure created due to neutralizing antibodies [46], etc. The infection rate reduced significantly across India after peaking in August 2020. However, India witnessed a ferocious ‘second wave’ of infection during February to June 2021, with the number of deaths several fold higher than that observed in the first wave [47]. This massive spread has mostly been attributed to the Delta variant, which has been shown to be associated with higher transmissibility rates than previously circulating Alpha and Kappa lineages [48, 49]. The Delta variant rapidly displaced all previously circulating viral lineages owing perhaps to its increased fitness [*R*_0_ (*R*_e_) 60–70 %] with viraemia 1000-fold higher than most previous lineages [49]. The increased adaptability of a pathogen virus under ‘waning’ immune pressure or partial immunization has been reported in other studies [50]. While other widely transmitted lineages such as Alpha emerged in other countries before vaccination programmes were implemented [6], the countrywide spread of Delta probably occurred during the implementation of vaccination in India. This suggests that higher mutation accumulation observed in this variant could be a result of increased selection pressure under a modified host microenvironment to which the virus was exposed. This suggestion assumes significance given the Delta variant’s ultrafast replication speeds which makes it detectable within 4 days after exposure [49]. Despite the preponderance of Delta during the second wave, the spread of specific virus lineage(s)/sub-lineages did exhibit differences in various geographical regions within India. Under these circumstances, it becomes imperative to study the evolution of the virus within a specific demography over an extended period.

From June 2021 onwards, the Delta variant itself was divided into several sub-lineages (based on Pangolin classification) but we have presented results mainly for the more frequently observed ones, namely AY.20, AY.39 and AY.44. The preferential occurrence of certain sub-lineages over others in different states within India and in different countries indicates a potential role of population-specific host genetic factors which might govern the favourable spread of one sub-lineage over another. However, we have not evaluated the association between host genetics and viral sub-lineages in this study. Although there are multiple reports of the association of a few S protein NVs (especially T478K, P681R and L452R) with viral transmissibility and immune escape [11 51], we have performed extensive analyses on the entire landscape of SARS-CoV-2 genomic NVs in this study. Although these NVs have been reported to occur in samples belonging to the Delta lineage (as documented in GISAID, and https://outbreak.info/situation-reports/delta), their association with vaccination breakthrough events was not reported, highighting the importance of our analysis. However, an absence of information on neutralization antibody levels in vaccinated individuals did make it difficult for us to establish a strong association with vaccination breakthrough cases. Nation-wide sero-surveys have suggested that sero-positivity or presence of neutralization IgG antibodies can exist even in unvaccinated individuals [52] (probably arising from natural infection). To the best of our knowledge, a comparison between sero-positive unvaccinated and vaccinated individuals for specific NV prevalence has not been performed. Also, keeping in mind the interpretation pitfalls that may be created due to the small size of vaccination breakthrough cases, it is important to validate the results presented here on a larger sample set, which we are currently pursuing. This work gains further significance in the context of recent reports that discuss the causal relationship between specific genomic variations and their potential to cause enhanced immune escape, even in individuals who have received vaccinations that are currently recognized by WHO [53–55].

NV cross-correlation analysis is a powerful tool to establish moderately or tightly linked genome-wide signatures. Our analyses revealed the complete footprint of genomic alterations affiliated with specific viral lineages. The cross-correlation analysis not only described the entire set of alterations associated with Delta and its sub-lineages, but also revealed several NVs that were completely mutually exclusive with those associated with previously circulating lineages. Another significant observation made possible through the cross-correlation analysis was the preferential co-occurrence of specific NVs in Delta sub-lineages but absent from Delta itself, which could not be deduced from linear analysis of NV timelines. The analyses also suggested greater enrichment of certain co-occurring NVs in vaccination breakthrough (compared to other) cases.

Previous studies have provided evidence on how iSNVs impart genomic plasticity [56] and direct viral genome evolution through inter-host transmission cycles [57, 58]. Due to lack of data on donor–recipient pairs and primary contacts (including family members) of infected individuals, we were unable to perform iSNV bottleneck estimation in this study. Moreover, despite having a substantial dataset size, the information output is hampered by lack of clinical information such as infection symptoms, hospitalization status, etc. Nevertheless, a few observations are worth highlighting. First, we did not detect a significant correlation between samples showing iSNVs and their vaccination status or their age or with a specific lineage (data not shown). Second, we identified specific iSNVs such as 25 907T in ORF3a that exhibited increased sample and allele frequencies with time. Interestingly, the 172V alteration generated from 25 907T has been shown to improve protein stability, owing to increased local hydrophobic interactions, in recent studies [59]. The stability of ORF3a plays a crucial role in its functionality as an apoptosis-inducing protein leading to cell death [60] and membrane rearrangement during SARS-CoV-2 infection [61].

Our iSNV analysis has potentially revealed an important S protein allele, namely 1101Y, where the ‘Y’ allele frequency showed an upward trend from April 2021 and was labelled as an NV as its frequency becamereached >50 %. Further, this NV was also preferentially associated with partially vaccinated samples. Interestingly, H1101Y, V1104L and T1117I are located between the two heptapeptide repeat sequences HR1 and HR2 within the S2 subunit of the S protein (Fig. S8). Earlier reports have suggested that both V1104L and H1101Y could increase local stability and alter the surface character of the S protein, thereby aiding favourable evolution of the virus [62, 63]. We therefore recommend including H1101Y and V1104L under active surveillance.

This study provides fresh insights into how the virus genome landscape has evolved over the duration of 19 months. Multiple factors related to host genetics, including co-morbidities and innate immune response, have been shown to play an important role in determining the evolution of virus variants [18]. However, since the current study is focused on samples obtained from the state of Telangana, we expect less host genetic variation among the patients analysed when compared to a pan-India study. The novelty of the study stems from its all all-inclusive approach and the identification of missense NVs in the context of cross-correlation and intra-host diversity analysis. We suggest mutation cross-correlation and iSNV analyses as two important tools for future studies targeting other geographical regions as well as a more recent timeline (including the emergence and rapid spread of Omicron, which nevertheless resulted in a much ‘milder’ [64] and subdued third wave across India; https://ourworldindata.org/covid-cases). More importantly, we laid greater emphasis on individual NVs rather than the lineages per se, given the recent and frequent ‘re-classifications’ of SARS-CoV-2 lineages by Pangolin [36]. Our study has facilitated a better understanding of how different aspects of virus genome dynamics are inter-linked. Future functional studies on important NVs identified in this study may reveal their possible role(s) in virus transmission and vaccine escape.

## Supplementary Data

Supplementary material 1

Supplementary material 2

Supplementary material 3

Supplementary material 4

Supplementary material 5

Supplementary material 6

Supplementary material 7
