# Supplementary material for: Assessing the evolution of SARS-CoV-2 lineages and the dynamic associations between nucleotide variations
Source: Access Microbiol. 2023 Jul 20;5(7):acmi000513.v3. doi: 10.1099/acmi.0.000513.v3 (PMC10436015; doi:10.1099/acmi.0.000513.v3)
Supplement: Supplementary material 1 [file acmi-5-513.v3-s001.pdf]

Supplementary figures

Figure S1 Timeline of major SARS-CoV-2 lineage distribution in Telangana, India and the world. (a) SARS-CoV-2 lineage timeline for Telangana, India. (b) Dominant lineages observed in other parts of India; states are pooled into distinct geographical regions viz. Northern (comprising of Rajasthan, Delhi, Uttar Pradesh, Haryana, Punjab, Uttarakhand, Himachal Pradesh, Ladakh, Jammu and Kashmir, Chandigarh), Western (comprising Gujarat, Maharashtra, Goa, Dadar &Nagar Haveli), Southern (comprising Andhra Pradesh, Telangana, Karnataka, Tamil Nadu, Kerala), Central (Madhya Pradesh, Chhattisgarh), Eastern (West Bengal, Bihar, Jharkhand, Odisha), North East (Mizoram, Tripura, Manipur, Nagaland, Assam, Arunachal Pradesh, Assam, Meghalaya, Sikkim). (c) Timeline of changing frequency of the Delta variant (blue) and total (grey) cases in Oceania, along with frequency change in the Delta sublineages (sequence contribution coming mostly from Australia; color key for lineages is same as in figure 1b). (d) (top) Distribution of lineages assigned to samples designated as vaccination breakthroughs (bottom) fraction of total lineages which were assigned to vaccination breakthrough cases (lineages which were present in <5 samples were excluded).

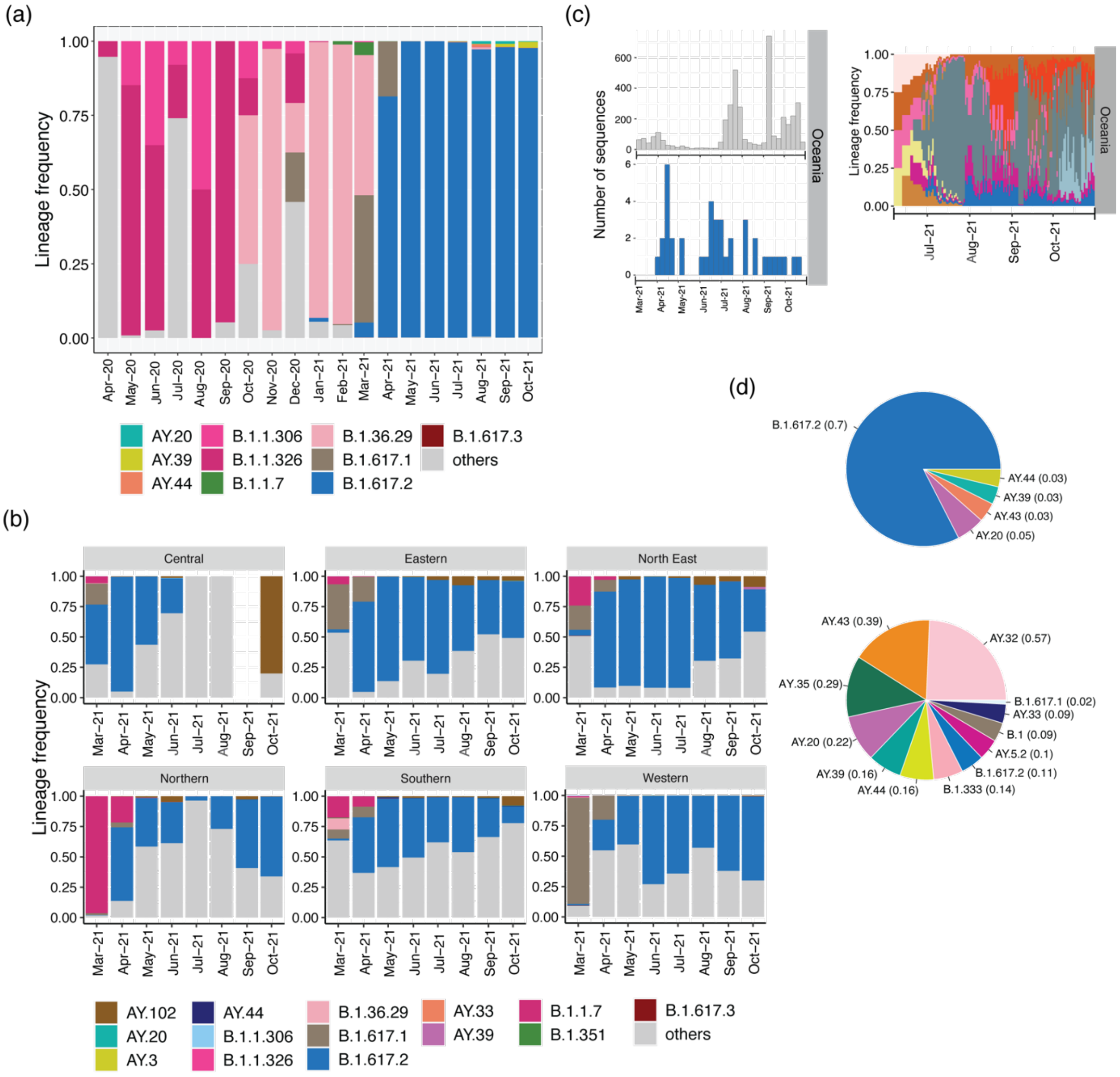
(a)
Lineage frequency
1.00
0.75
0.50
0.25
0.00
Apr–20
May–20
Jun–20
Jul–20
Aug–20
Sep–20
Oct–20
Nov–20
Dec–20
Jan–21
Feb–21
Mar–21
Apr–21
May–21
Jun–21
Jul–21
Aug–21
Sep–21
Oct–21
AY.20
AY.39
AY.44
B.1.1.306
B.1.1.326
B.1.1.7
B.1.36.29
B.1.617.1
B.1.617.2
B.1.617.3
others
(b)
Central
Eastern
North East
Northern
Southern
Western
Lineage frequency
Mar–21
Apr–21
May–21
Jun–21
Jul–21
Aug–21
Sep–21
Oct–21
AY.102
AY.20
AY.3
AY.44
B.1.1.306
B.1.1.326
B.1.36.29
B.1.617.1
B.1.617.2
AY.33
AY.39
B.1.1.7
B.1.351
B.1.617.3
others
(c)
Number of sequences
Oceania
Lineage frequency
Jul–21
Aug–21
Sep–21
Oct–21
(d)
B.1.617.2 (0.7)
AY.44 (0.03)
AY.39 (0.03)
AY.43 (0.03)
AY.20 (0.05)
AY.43 (0.39)
AY.32 (0.57)
AY.35 (0.29)
B.1.617.1 (0.02)
AY.33 (0.09)
B.1 (0.09)
AY.5.2 (0.1)
B.1.617.2 (0.11)
B.1.333 (0.14)
AY.44 (0.16)
AY.39 (0.16)
AY.20 (0.22)

Figure S2: Profile of all SARS-CoV-2 NVs identified in >3% samples from Telangana, India. The position of different proteins are shown on top (nsp boundaries in ORF1a and ORF1b are indicated as small vertical black bars).

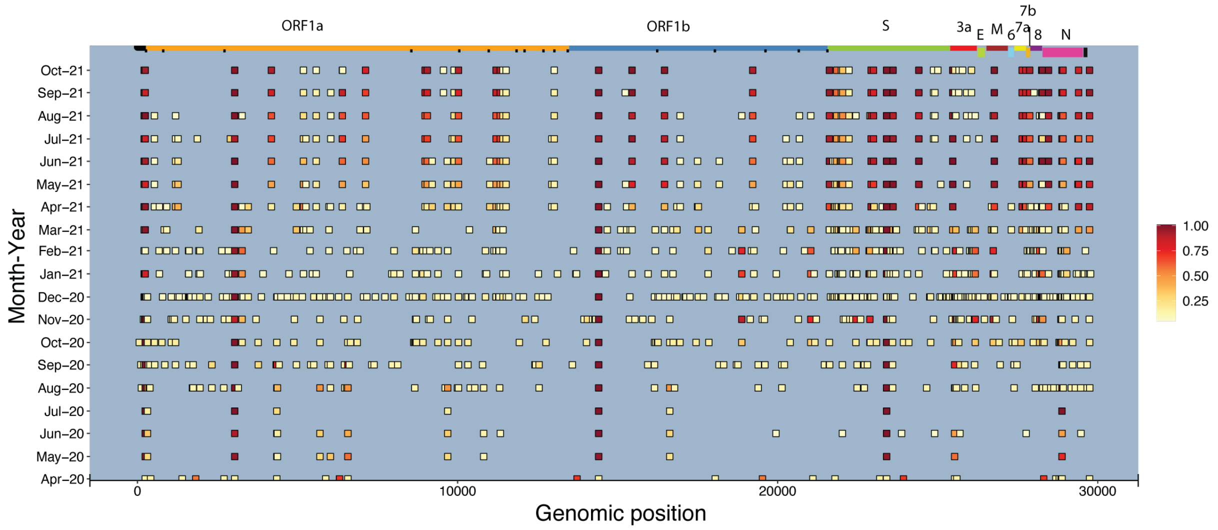

Figure S3: Frequency of all missense NVs (found in >3% of samples) in different virus lineages. The lineages on the y-axis are arranged in the order (bottom to top) they appeared in the population. Color key of frequency is same as in Figure 2.

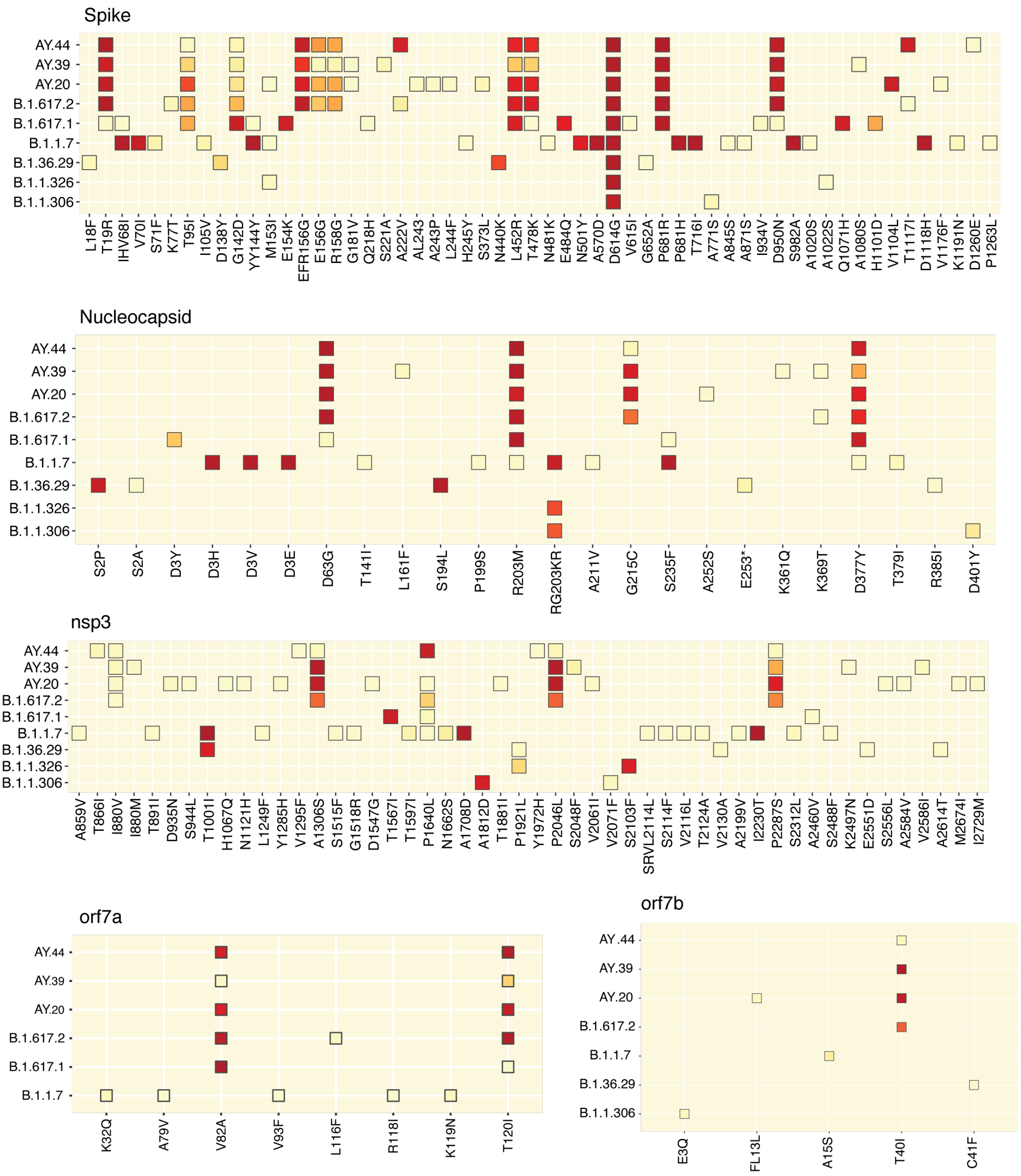

Figure S4: Timeline figure indicating month-wise (a, c) and lineage-wise (b, d) distribution of amino acid variants in ORF3a (a, b) and nsp12 (c, d). Colour scheme is same as shown in Figure 2.

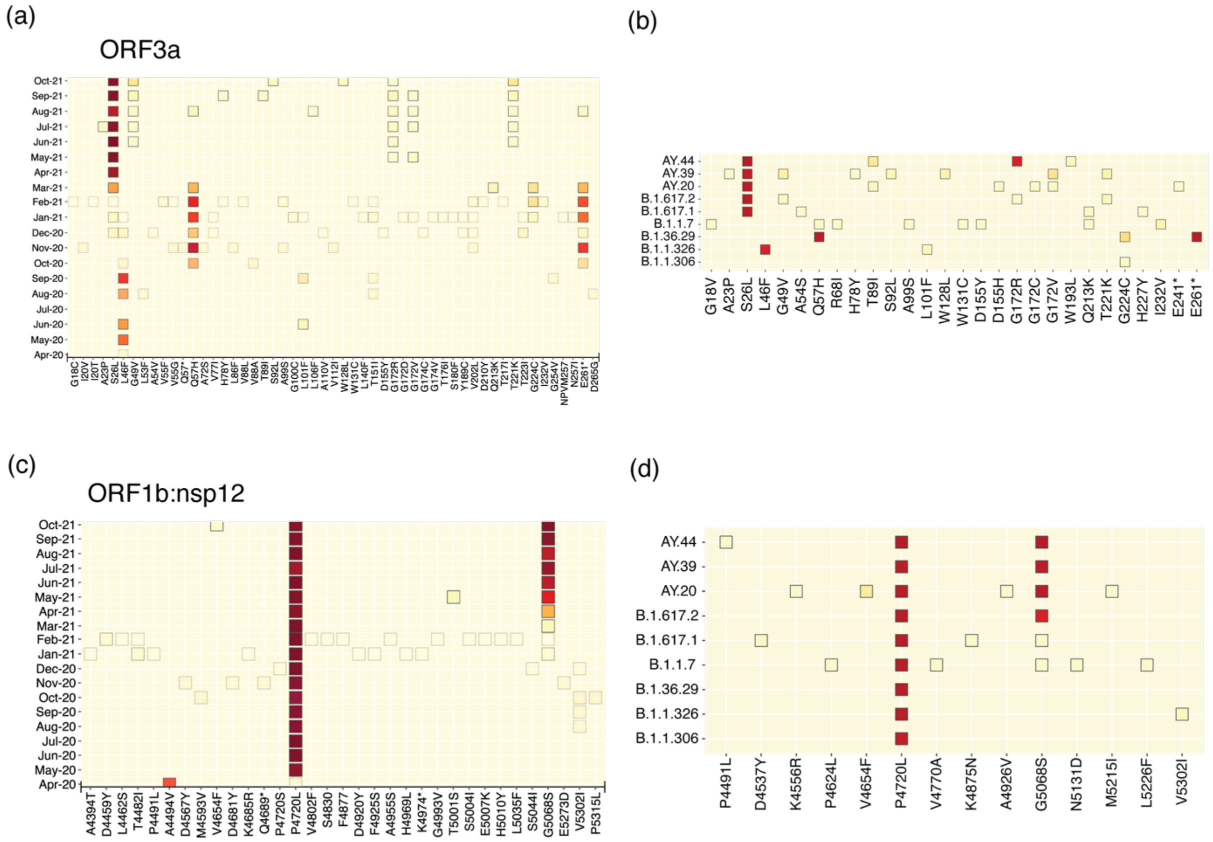

Figure S5: Frequency of amino acid changes found in >3% of all vaccinated samples.

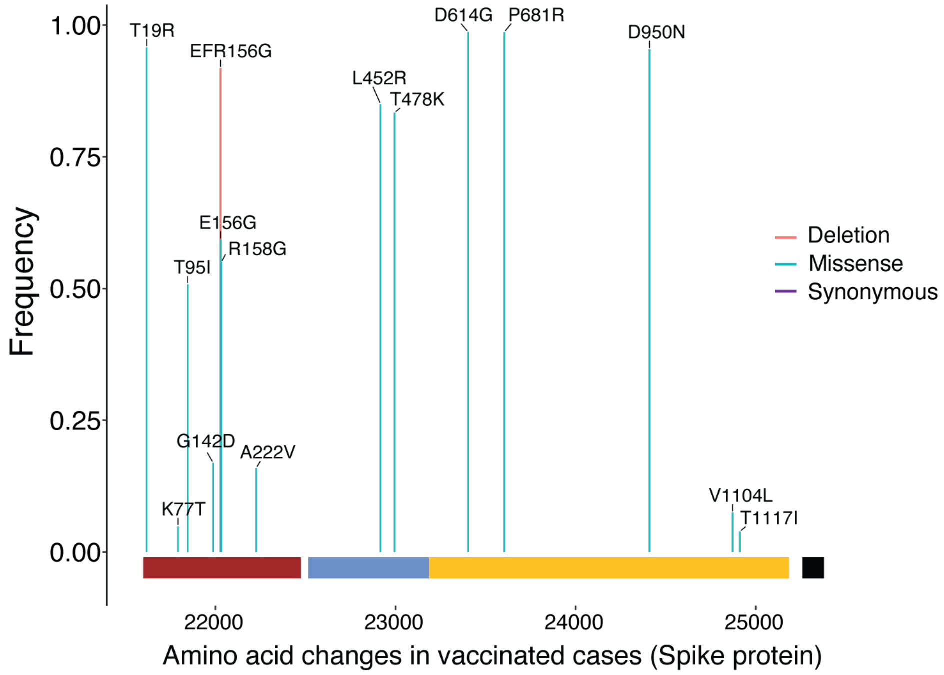

Figure S6: Comparative NV cross-correlation analysis in vaccinated and non-vaccinated cases. (a) Pairwise NV cross-correlation between all S protein missense NVs found in >3% of samples in vaccination breakthrough cases. (b) NV cross-correlation calculated from a random subset of 350 samples from March 2021 onwards consistent with the timeline of vaccination breakthrough samples. Colour scheme is same as in Figure 3c.

(a)

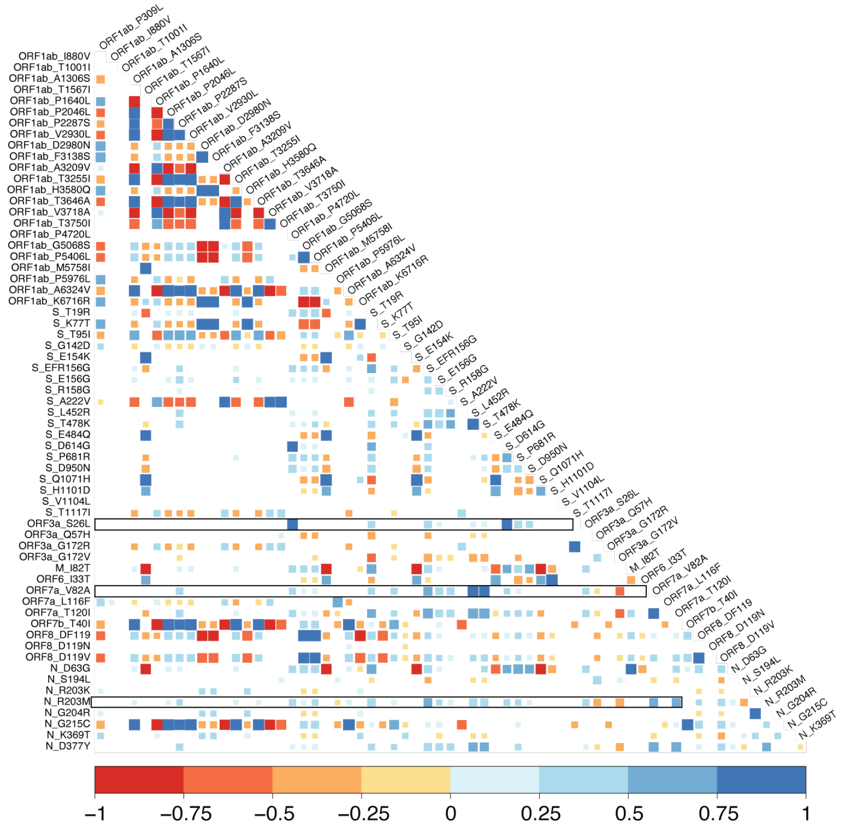
ORF1ab_P309L
ORF1ab_I880V
ORF1ab_T1001I
ORF1ab_A1306S
ORF1ab_T1567I
ORF1ab_P1640L
ORF1ab_P2046L
ORF1ab_P2287S
ORF1ab_V2930L
ORF1ab_D2980N
ORF1ab_F3138S
ORF1ab_A3209V
ORF1ab_T3255I
ORF1ab_H3580Q
ORF1ab_T3646A
ORF1ab_V3718A
ORF1ab_T3750I
ORF1ab_P4720L
ORF1ab_G5068S
ORF1ab_P5406L
ORF1ab_M5758I
ORF1ab_P5976L
ORF1ab_A6324V
ORF1ab_K6716R
S_T19R
S_K77T
S_T95I
S_G142D
S_E154K
S_EFR156G
S_E156G
S_R158G
S_A222V
S_L452R
S_T478K
S_E484Q
S_D614G
S_P681R
S_D950N
S_Q1071H
S_H1101D
S_V1104L
S_T1117I
ORF3a_S26L
ORF3a_Q57H
ORF3a_G172R
ORF3a_G172V
M_I82T
ORF6_I33T
ORF7a_V82A
ORF7a_L116F
ORF7a_T120I
ORF7b_T40I
ORF8_DF119
ORF8_D119N
ORF8_D119V
N_D63G
N_S194L
N_R203K
N_R203M
N_G204R
N_G215C
N_K369T
N_D377Y
−1
−0.75
−0.5
−0.25
0
0.25
0.5
0.75
1

(b)

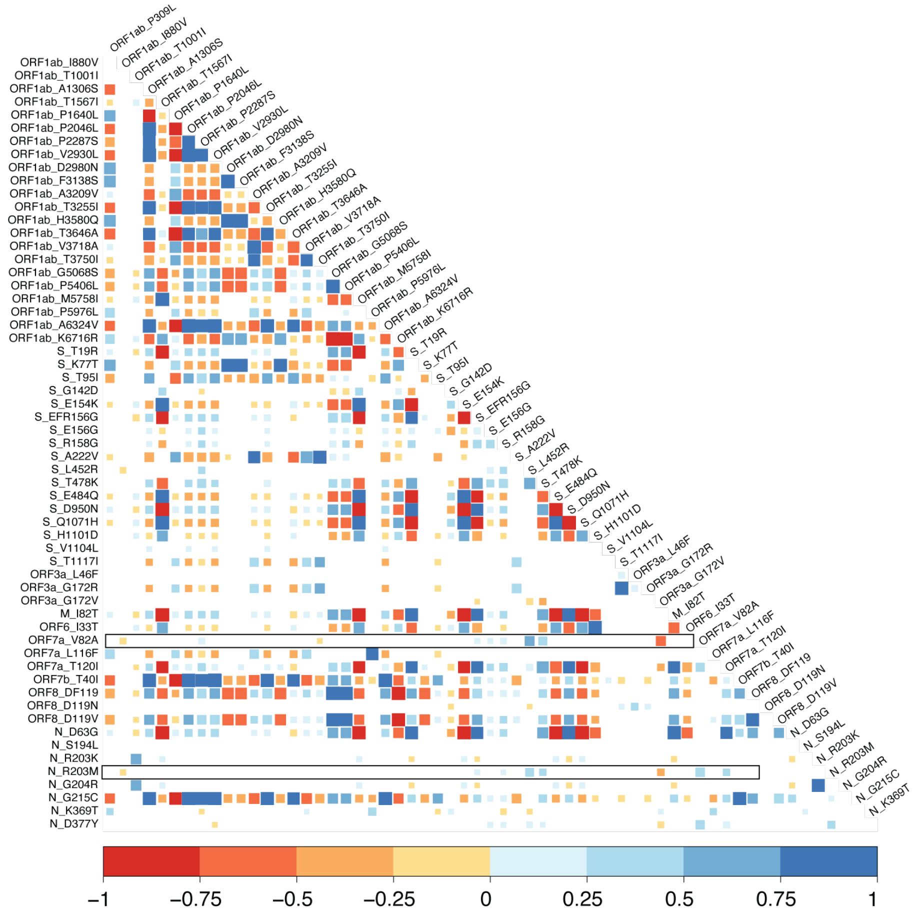
ORF1ab_P309L
ORF1ab_I880V
ORF1ab_T1001I
ORF1ab_A1306S
ORF1ab_T1567I
ORF1ab_P1640L
ORF1ab_P2046L
ORF1ab_P2287S
ORF1ab_V2930L
ORF1ab_D2980N
ORF1ab_F3138S
ORF1ab_A3209V
ORF1ab_T3255I
ORF1ab_H3580Q
ORF1ab_T3646A
ORF1ab_V3718A
ORF1ab_T3750I
ORF1ab_G5068S
ORF1ab_P5406L
ORF1ab_M5758I
ORF1ab_P5976L
ORF1ab_A6324V
ORF1ab_K6716R
S_T19R
S_K77T
S_T95I
S_G142D
S_E154K
S_EFR156G
S_E156G
S_R158G
S_A222V
S_L452R
S_T478K
S_E484Q
S_D950N
S_Q1071H
S_H1101D
S_V1104L
S_T1117I
ORF3a_L46F
ORF3a_G172R
ORF3a_G172V
M_I82T
ORF6_I33T
ORF7a_V82A
ORF7a_L116F
ORF7a_T120I
ORF7b_T40I
ORF8_DF119
ORF8_D119N
ORF8_D119V
N_D63G
N_S194L
N_R203K
N_R203M
N_G204R
N_G215C
N_K369T
N_D377Y
−1
−0.75
−0.5
−0.25
0
0.25
0.5
0.75
1

Figure S7: Sample wise distribution of all NVs and alternative/minor alleles generated as a results of iSNVs at genomic loci with shared iSNV and NV (only alterations, either major or minor, present in > 5 samples are shown; all loci with > 25 samples are labelled)

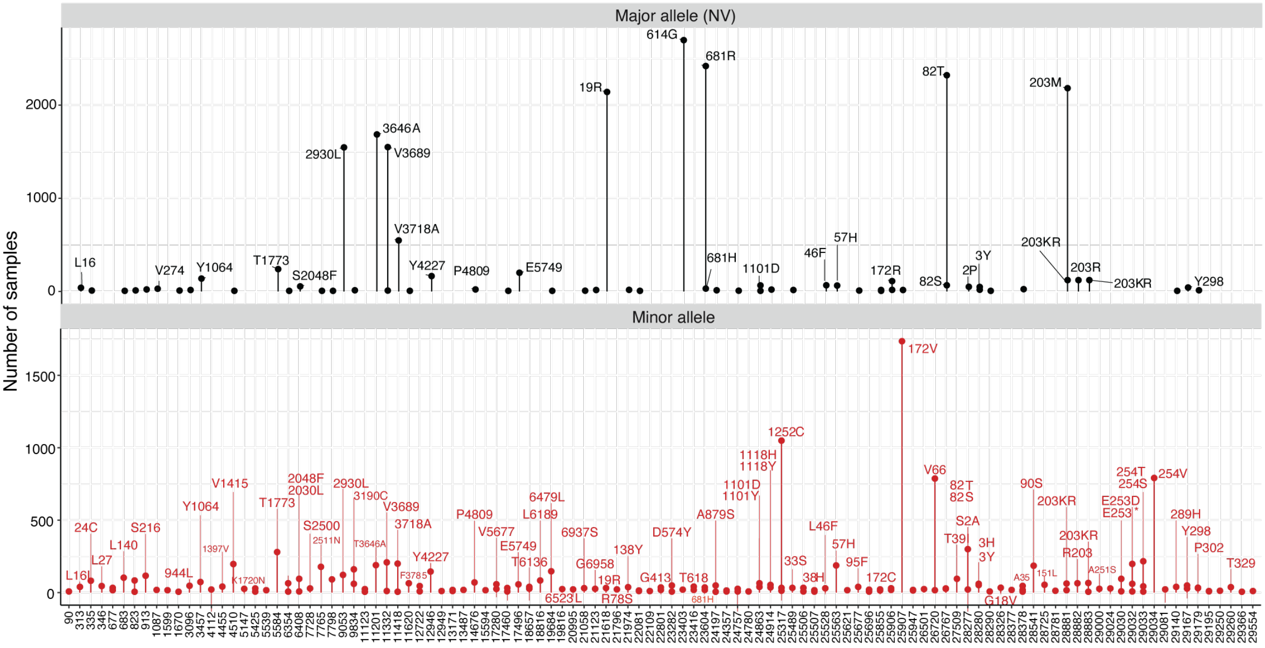

Figure S8: Location of V1104, H1101, and T1117 mapped on the S protein structure.

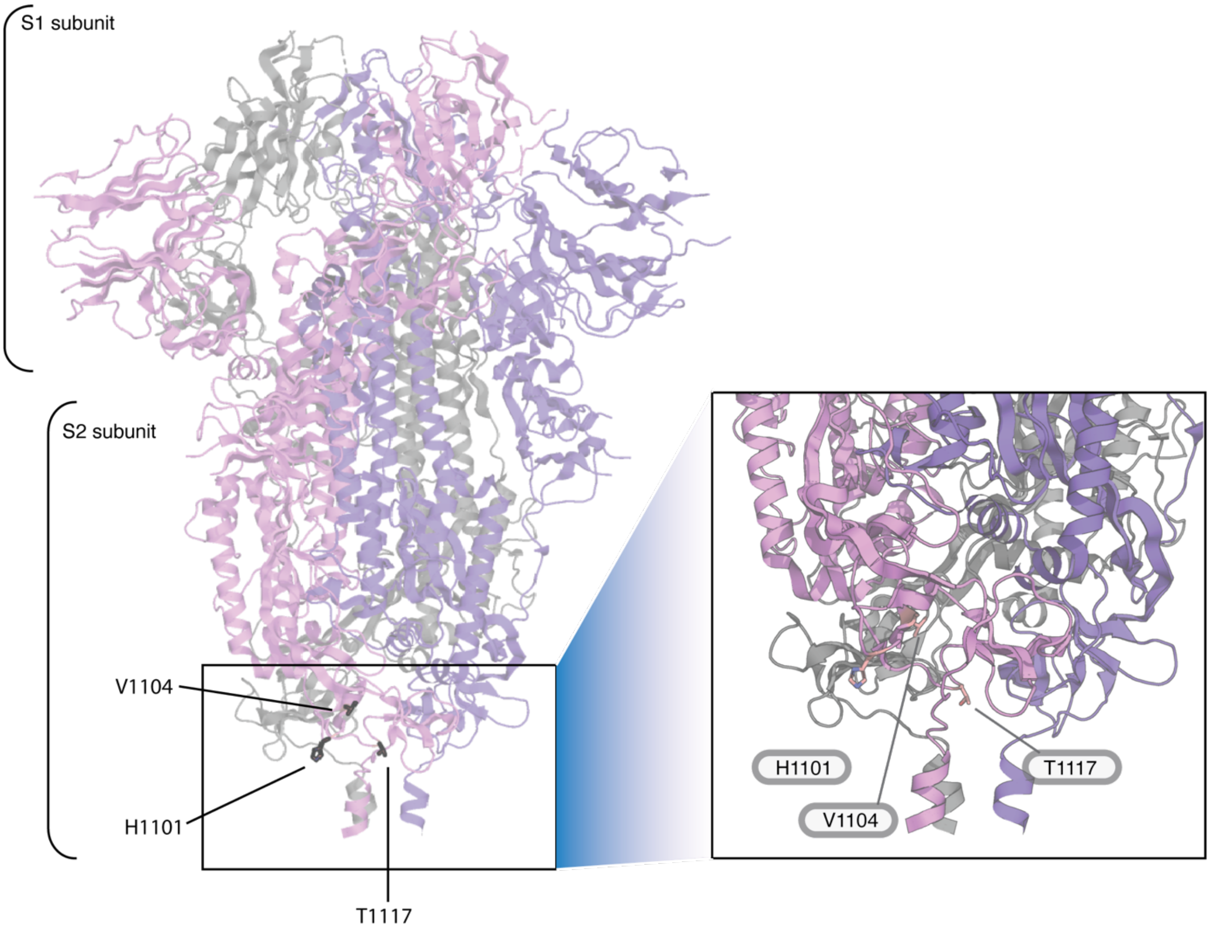

Supplementary data

Table S1A: Metadata associated with all 3543 samples analyzed in this work.

Table S1B: Metadata of vaccination breakthrough samples from Tamil Nadu.

Table S1C: Metadata of vaccination breakthrough samples from Uttar Pradesh.

Table S2: List of accession IDs of all the sequences submitted to GISAID.

Table S3: Distribution of all lineages observed in the population till October 2021.

Table S4: Odds ratio point estimates and associated p-values for the NVs found in >5% of samples.

Table S5: Ct values (for RdRp) of all samples showing presence of >9 iSNVs.

Table S6: Distribution of the number of iSNV sites per gene, normalized by the gene size
